# SPSS 31.0 Versus GPT-5.5: Performance Across Core Epidemiological and Non-parametric Statistics in Medicine and Applied Health Sciences

**DOI:** 10.7759/cureus.115164

**Published:** 2026-08-25

**Authors:** Frederick F Strale, Rachael German, VeraLucia Mendes-Kramer, Steven B Hammer, Michal Hajek, Walter P Fulbright, Robert Ehrman, Robert Sherwin

**Affiliations:** 1 Applied Health Sciences, Eugene Applebaum College of Pharmacy and Health Sciences, Wayne State University, Detroit, USA; 2 Mortuary Science, Eugene Applebaum College of Pharmacy and Health Sciences, Wayne State University, Detroit, USA; 3 Medical and Exercise Physiology, Indian River State College, Fort Pierce, USA; 4 Hyperbaric Medicine, Centre for Hyperbaric Medicine of Faculty of Medicine, University of Ostrava and Ostrava City Hospital, Ostrava, CZE; 5 Georgia Center for Continuing Education, University of Georgia, Athens, USA; 6 Emergency Medicine, Wayne State University Detroit Medical Center, Detroit, USA; 7 Emergency Medicine, School of Medicine, Wayne State University, Detroit, USA

**Keywords:** ai-assisted statistics, alignment, chatgpt, ibm spss, large language models

## Abstract

Background

The growing adoption of artificial intelligence (AI) technologies, including large language models (LLMs), such as ChatGPT (OpenAI, San Francisco, CA) and many others, has expanded their role in statistical analysis and scientific research workflows. Despite this increasing use, conventional statistical software, such as IBM SPSS (IBM Corp., Armonk, NY), remains the accepted reference standard because of its established methodological precision, transparency, and reproducibility. Consequently, it is important to determine whether statistical results generated by LLMs achieve comparable levels of accuracy and consistency. As these systems undergo continual refinement, independent replication, comparative validation, and continuation studies are necessary to assess whether newer model generations produce findings that reliably correspond with those obtained using established statistical methodologies.

Methodology

Thirteen statistical procedures commonly used in clinical, medical, epidemiological, and applied health sciences research were evaluated using authentic datasets derived from previously published peer-reviewed studies and undergraduate applied health sciences coursework. Analyses included epidemiological tests, nonparametric analyses, chi-square analyses, univariable and multivariable logistic regression, Kaplan-Meier survival analysis, Cox proportional hazards regression, and measures of nominal association. For each procedure, dataset and variable names were copied directly from SPSS 31.0 data files and analyzed in GPT-5.5 using standardized prompts. AI-generated results were then compared directly with SPSS 31.0 outputs to evaluate computational agreement, methodological consistency, and inferential concordance across all statistical procedures.

Results

GPT-5.5 demonstrated a high degree of agreement with SPSS 31.0 across all statistical procedures evaluated, including risk and odds ratios, Wilcoxon signed-rank, Mann-Whitney U, Kruskal-Wallis, Friedman's test, one-way and two-way chi-square, binary and multivariable logistic regression, Kaplan-Meier survival analysis, Cox proportional hazards model, and phi and Cramer's V. Most analyses produced identical test statistics, effect estimates, confidence intervals, and inferential conclusions. Minor numerical differences were observed only in selected approximation statistics, including standardized *Z* values, Wald statistics, and Cox regression parameter estimates, and were attributable to expected implementation differences in optimization routines, convergence criteria, tie corrections, or reporting conventions. Importantly, none of these differences altered statistical significance, effect interpretation, or the overall scientific conclusions.

Conclusions

GPT-5.5 demonstrated excellent statistical consistency with SPSS 31.0 across a broad range of statistical analyses, producing complete or near-complete agreement in nearly all computational and inferential outcomes. Minor numerical differences were limited to implementation-specific approximations and did not affect statistical significance or substantive interpretation. These findings support the use of GPT-5.5 as a reliable adjunct for statistical verification, interpretation, and research support while reinforcing that independent methodological oversight and validation with established statistical software remain essential for scientific research.

## Introduction

Artificial intelligence (AI) is rapidly reshaping the conduct of scientific research by assisting with data analysis, statistical interpretation, and methodological decision-making. Among these technologies, large language models (LLMs), including ChatGPT (OpenAI, San Francisco, CA), have shown the capacity to perform a wide range of statistical tasks, generate analytical interpretations, and produce written summaries that closely resemble those prepared by experienced researchers [1-5]. As the adoption of these systems continues to expand across biomedical and clinical research, an important question has emerged: Can AI-generated statistical analyses provide results that are sufficiently accurate, reproducible, and dependable to support evidence-based research and clinical decision-making?

Despite rapid advances in AI, established statistical software packages, including IBM SPSS (IBM Corp., Armonk, NY), SAS (SAS Institute, Cary, NC), R (R Foundation for Statistical Computing, Vienna, Austria), Stata (StataCorp LLC, College Station, TX), and others, continue to serve as the accepted reference standard for statistical analyses in medicine and the health sciences. These platforms rely on validated computational algorithms, clearly documented analytical procedures, and stable software versions that facilitate transparency and reproducibility. By comparison, LLMs are continually refined through updates to their underlying architectures, training processes, and optimization methods. Consequently, statistical results generated by these models may differ between software versions or even across repeated interactions using similar prompts, underscoring the importance of systematically evaluating their analytical consistency [2,6-9].

Investigations comparing conventional statistical software with AI-generated analyses examined the agreement between IBM SPSS and ChatGPT-4 and o3-mini models [10]. Findings demonstrated strong concordance for descriptive statistics and several commonly used inferential procedures, while also identifying meaningful differences in more complex analytical methods, including factorial analysis of variance (ANOVA) and multivariate analysis of variance (MANOVA). These observations emphasized both the promise of LLM-assisted statistical analysis and the need for continued assessment of their performance in advanced statistical applications [2,9-11].

In subsequent analyses, the researchers reported a high degree of concordance between the two platforms for numerous routinely applied statistical methods, including descriptive analyses, correlation coefficients, regression models, t-tests, and one-way ANOVA. Nevertheless, larger differences were observed for several advanced analytical techniques, most notably factorial ANOVA, MANOVA, and repeated-measures ANOVA [12].

Based on these findings, the researchers concluded that ChatGPT-5.5 has potential value as a supplementary resource for statistical interpretation and exploratory analyses. However, they emphasized that statistical results generated by AI should be independently verified and interpreted judiciously, particularly in studies informing clinical practice. Conventional statistical software, together with appropriate methodological expertise, continues to be indispensable for confirmatory statistical analyses and evidence-based clinical research [12].

As LLMs evolve through frequent model updates, replication and continuation studies have become increasingly important for evaluating their statistical performance over time [7,10,12]. Unlike conventional statistical software, whose algorithms and version changes are generally stable and thoroughly documented, LLMs may exhibit changes in performance resulting from architectural refinements, updated training data, or reinforcement learning modifications, a phenomenon commonly described as model drift [8]. Accordingly, repeated validation studies are essential not only for confirming the reproducibility of previous findings but also for determining whether successive generations of LLMs demonstrate greater statistical accuracy, methodological consistency, and agreement with established analytical standards [2,7,9].

Additional investigations are warranted to examine the performance of LLMs across a broader range of sophisticated statistical methodologies in medicine and the applied health sciences. Future studies should include analyses such as epidemiologic modeling, non-parametric analyses, univariable and multivariable logistic regression, and survival analyses [11,12]. Expanding comparative evaluations to include established statistical computing platforms such as SPSS, R, SAS, Stata, and Python-based analytical ecosystems would provide a more comprehensive understanding of the capabilities and limitations of AI-assisted statistical analysis. Furthermore, repeated evaluations across future generations of LLMs will be important for monitoring model drift and determining whether ongoing model refinements improve the stability, reproducibility, and accuracy of AI-generated statistical results over time [11,12].

Research objective

The current study further evaluates the statistical performance of GPT-5.5 (OpenAI) through direct comparisons with IBM SPSS Statistics 31.0 using empirical research datasets, published textbook datasets, and simulated/instructional datasets. To provide a comprehensive assessment, analyses were conducted across a broad spectrum of statistical techniques ranging from basic to highly advanced procedures in epidemiology, medicine, and the applied health sciences. These include risk and odds ratios, the Wilcoxon signed-rank test, the Mann-Whitney U test, the Kruskal-Wallis test, Friedman's test, one-way and two-way chi-square, univariable and multivariable logistic regression, Kaplan-Meier survival analysis with the Mantel-Cox (log-rank) test, the Cox proportional hazards model, and phi and Cramer's V. By expanding the scope of previous comparative evaluations, this investigation aims to further characterize the capabilities and limitations of AI-assisted statistical analysis while contributing evidence to guide the responsible incorporation of LLM-generated statistical outputs into scientific research and quantitative analytical workflows [1,4,10-12].

## Materials and methods

Data collection

Between July 4 and July 13, 2026, this investigation synthesized quantitative evidence from three primary sources: peer‑reviewed studies conducted across multiple research sites, published doctoral dissertations, and instructional datasets developed for the Advanced Statistics for Applied Health Sciences course (AHS2030) at Wayne State University [13-18]. These datasets span a broad temporal window (2012-2026), providing a consistent and representative foundation for evaluating trends in health and biologically oriented research.

Across studies, the data focused on variables mostly central to clinical and therapeutic inquiry, including descriptive health indicators, associations among biological and behavioral measures, and both univariate and multivariate group comparisons. Independent variables were systematically coded to support structured analyses of treatment conditions, intervention phases, and categorical groupings, such as those used in 2×2 contingency table designs. Corresponding dependent variables captured measurable physiological or behavioral outcomes relevant to epidemiology, medicine, and applied health sciences [13-18].

One dataset incorporated a repeated‑measures framework, enabling the examination of within‑subject change across multiple observation points. This longitudinal structure enhanced statistical power and allowed for a more precise assessment of treatment effects over time [14].

AI‑assisted statistical procedures were performed using ChatGPT Plus (OpenAI), powered by the GPT‑5.5 model, during the period of July 4-13, 2026. The analytic scope encompassed a broad range of epidemiological and inferential techniques, including risk ratios (RRs) and odds ratios (ORs), Wilcoxon signed-rank test, Mann-Whitney U, Kruskal-Wallis, Friedman’s test, and one‑way and two‑way chi‑square analyses. Additional procedures included univariable and multivariable logistic regression, Kaplan-Meier survival analysis with Mantel-Cox testing, Cox proportional hazards modeling, and phi and Cramer's V.

All reference analyses were conducted in SPSS 31.0 following established methodological standards and best‑practice guidelines [6], with an alpha level of 0.05 applied uniformly across hypothesis tests. Assumptions relevant to each procedure were evaluated in both SPSS 31.0 and GPT‑5.5. Missing data were handled in SPSS 31.0 using the default “Exclude cases analysis by analysis” option, and equivalent case‑wise exclusion was mirrored in GPT‑5.5. Consequently, any variation in sample size across analyses reflects differences in variable availability rather than discrepancies in missing‑data processing between platforms [3,6].

Datasets and statistical outputs were maintained within SPSS 31.0 and served as the foundation for direct comparison with GPT‑5.5 results [3,6]. For each statistical procedure, the corresponding SPSS 31.0 dataset (.sav) was accessed through the GPT-5.5 interface together with a standardized, analysis-specific prompt. GPT-5.5 utilized the SPSS 31.0 dataset that was already open within the user's SPSS 31.0 session and performed the requested statistical analysis using the variables specified in the prompt. The resulting statistical output was then compared directly with the corresponding output generated independently by SPSS 31.0. Variable names were retained exactly as they appeared in the original SPSS 31.0 datasets to ensure methodological consistency between the two analytical workflows.

As an internal quality-control measure, selected SPSS 31.0 analyses were periodically repeated using the same datasets and statistical procedures to confirm output stability and reproducibility. These repeat analyses consistently produced identical results and served solely to verify analytical consistency. Accordingly, because they did not constitute independent analyses or generate new findings, they are not reported separately.

Methodological equivalence was preserved by using identical datasets, without any post‑import transformations, recoding, or preprocessing, for both SPSS 31.0 and GPT‑5.5 [13-18]. Data integrity was ensured through prior logic checks, verification of variable types, and consistent formatting during the original SPSS 31.0 data‑entry and cleaning processes. Numeric and categorical variables were retained in their correct formats, and clear variable labeling supported accurate interpretation. As all validation procedures had been completed during the initial SPSS 31.0 workflow, the datasets used in this study were already confirmed to be high‑quality and analytically reliable [13-18].

A final methodological consideration concerns reproducibility in AI‑assisted research [7]. The GPT‑5.5 interface does not provide model‑specific technical identifiers such as build numbers, weight versions, checksums, or application programming interface (API) revision details [3]. As a result, documenting the subscription tier, model designation, and date range of access represents the highest level of reproducibility currently achievable when using the consumer‑facing ChatGPT Plus platform. These details are reported here in accordance with emerging recommendations for transparent AI‑methods reporting and to facilitate future replication and comparative methodological studies [12].

Statistical protocols

Thirteen fundamental epidemiological and nonparametric statistical methodologies were used to characterize datasets commonly encountered in clinical medicine, epidemiology, and the applied health sciences. The analytical procedures below provide a framework for quantifying associations and evaluating group differences, thereby establishing the foundation for ongoing inferential analyses and evidence-based interpretation.

Risk Ratio (Relative Risk)

The risk ratio (relative risk) is an epidemiological measure that compares the probability of an outcome occurring in an exposed group with the probability of the same outcome in an unexposed group. It quantifies the magnitude of association between an exposure and an outcome and is commonly used in cohort studies and randomized clinical trials.

Odds Ratio

The odds ratio is an epidemiological measure that compares the odds of an outcome occurring in one group relative to another. It is the primary measure of association in case-control studies and logistic regression analyses and provides an estimate of the strength and direction of the relationship between an exposure and a binary outcome.

Wilcoxon Signed-Rank Test

The Wilcoxon Signed-Rank test is a nonparametric alternative to the paired t-test used to compare two related (pretest-posttest) or repeated measurements when the assumption of normality is violated. It evaluates whether the median difference between paired observations differs significantly from zero.

Mann-Whitney U Test

The Mann-Whitney U test is a nonparametric alternative to the two independent-sample t-test that compares the distributions of a continuous or ordinal outcome between two independent groups. It is particularly appropriate when data are skewed or measured on an ordinal scale.

Kruskal-Wallis Test

The Kruskal-Wallis test is a rank-based nonparametric alternative to one-way ANOVA used to compare three or more independent groups. It determines whether at least one group differs significantly from the others without assuming normally distributed data.

Friedman's Test

Friedman's test is a nonparametric alternative to repeated-measures ANOVA for comparing three or more related conditions or time points. It evaluates whether median rankings differ across repeated measurements within the same participants.

One-Way Chi-Square Analysis (Goodness-of-Fit Test)

The one-way chi-square (goodness-of-fit) test evaluates whether the observed frequencies of a single categorical variable differ significantly from a specified or expected distribution. It is commonly used to determine whether observed proportions conform to theoretical expectations or established population distributions.

Two-Way Chi-Square Analysis (Test of Independence)

The two-way chi-square (test of independence) evaluates whether a statistically significant association (relationship) exists between two categorical variables by comparing observed and expected frequencies within a contingency table. It is widely used to assess whether the distribution of one categorical variable differs according to the levels of another variable.

Univariable Logistic Regression

Univariable logistic regression evaluates the association between a single predictor variable and a binary outcome by estimating the corresponding odds ratio and its confidence interval. It is commonly used to identify variables that may be associated with the outcome before multivariable modeling.

Multivariable Logistic Regression

Multivariable logistic regression simultaneously examines the independent effects of multiple predictor variables on a binary outcome while adjusting for potential confounding factors. By estimating adjusted odds ratios and corresponding confidence intervals, it identifies predictors that remain significantly associated with the outcome after controlling for the influence of other covariates.

Kaplan-Meier Survival Analysis With Mantel-Cox (Log-Rank) Test

Kaplan-Meier survival analysis estimates the probability of remaining event-free over time while accounting for censored observations. The Mantel-Cox (log-rank) test compares survival distributions between groups to determine whether statistically significant differences exist.

Cox Proportional Hazards Model

The Cox proportional hazards model is a semiparametric regression technique used to examine the effects of multiple predictors on time-to-event outcomes. It estimates hazard ratios while adjusting for covariates and assumes that hazard ratios remain proportional over the duration of follow-up.

Phi & Cramer's V

Phi measures the strength of association between two binary variables, while Cramer's V adjusts for table size. The value always falls between 0 and 1, making it a stable measure of association for any crosstabulation.

Data prompts for GPT-5.5

Data prompts for GPT-5.5 are listed in Table 1.

**Table 1:** Test statistic and data prompts for GPT-5.5.

Test statistic	Data prompts for GPT-5.5
Crosstabulations, risk and odds ratios	Please assemble a Crosstabulation Table, then compute a Risk Ratio and an Odds Ratio for the variables Smoking and LungCancer in this “Risk & Odds Data.sav” SPSS dataset
Wilcoxon signed-rank test	Please compute a Wilcoxon Signed-Rank Test from the variables PreHBOTGCS and PostHBOTGCS from the “Czechoslovakian data.sav” SPSS dataset
Mann-Whitney U	Please compute a Mann-Whitney U test from the variables Group and Q1_1 from the “Lou Kramer’s Dissertation Data #2.sav” SPSS dataset
Kruskal-Wallis	Please run a Kruskal-Wallis Test from the variables Group and Q1_1 from the “Lou Kramer’s Dissertation Data #2.sav” SPSS dataset
Friedman’s test	Please run a Friedman’s Test from the variables WellBeingScoreAtDischarge, WellBeingat3MonthsScore, and WellBeingat6MonthsScore from the “Stroke & Heart Attack Data.sav” SPSS dataset
One-way chi-square	Please compute a One-Way Chi Square with the variable Age_Binnes from the “Blood Pressure Complete Set n=236(1).sav” SPSS dataset
Two-way chi-square	Please compute a Two-Way Chi Square (Test of Independence) with the Age-Binnes, and GENDER variables from the “Blood Pressure Data Complete Set n=236(1).sav” SPSS dataset
Univariable logistic regression	Please run a Univariable Binary Logistic Regression with GENDER as the dependent variable and HR_pre_Sup as the independent variable from the dataset “Blood Pressure Data Complete Set n=236(1).sav” SPSS dataset
Multivariable logistic regression	Please run a Multivariable Binary Logistic Regression with GENDER as the dependent variable and HR_post_Sup and HR_post_std as the independent variables from the dataset “Blood Pressure Data Complete Set n=236(1).sav” SPSS dataset
Kaplan-Meier survival	Please run a Kaplan-Meier Survival Analysis looking at Time to Nausea Relief, with these Time2, Treatment2, and Status2 variables from the dataset “Survival Analysis Data(1).sav” SPSS dataset
Cox proportional hazards model	Please run a Cox Proportional Hazards Model with the variables Treatment2 and Ginger as predictor variables from the “Survival Analysis Data(1).sav” SPSS dataset
Phi and Cramer’s V	Please run a Phi & Cramer’s V on the variables JobSatf2 and FieldofStudy3 with the “Pat Fulbright Dissertation Data.sav” SPSS dataset

Ethics board approvals

The “Czechoslovakian Data.sav” employed a retrospective design; therefore, formal ethical approval was not required [16]. Under Czech Republic legislation, research projects that do not meet the legal definition of a clinical trial involving a medicinal product or medical device are exempt from ethics committee review, as stipulated in Act No. 378/2007 Coll. (§53) on medicinal products. The relevant ethics committee was consulted, and its determination is provided in the Supplementary Materials. As part of the informed consent process for hyperbaric oxygen therapy (HBOT) at our institution, patients authorize the retrospective review of their medical records and the anonymous use of health data for scientific and educational purposes [16].

The “Lou Kramer’s Dissertation Data #2.sav” was conducted in accordance with established ethical principles governing human participant research and received approval from the Institutional Review Board (IRB) at Wayne State University, Detroit, Michigan (Approval No. IRB-23-01-5400; March 21, 2023). Prior to enrollment, all participants were fully informed of the study's objectives, procedures, and potential risks and benefits, and written informed consent was obtained from everyone before participation and data collection commenced [15].

The "Pat Fulbright Dissertation Data.sav" received ethical approval from the Institutional Review Board (IRB) of Trident University under IRB Protocol #1080. The study was approved in accordance with the federal regulations governing the protection of human research participants (45 CFR 46.111). Following IRB approval, authorization was granted to commence data collection immediately, and annual continuing review was not required while the study remained ongoing under the approved protocol. All research procedures were conducted in accordance with the approved IRB protocol and applicable ethical standards for research involving human participants [17].

The "Blood Pressure Data Complete Set n=236(1).sav" received approval from the IRB at Indian River State College (IRSC) in Fort Pierce, Florida, in 2013. All participants provided written informed consent before study enrollment and participation in the ultramarathon events, in accordance with established ethical standards for research involving human participants [14].

The "Stroke & Heart Attack Data (1).sav" was derived from Statistics for the Health Sciences: A Non-Mathematical Introduction [13], where it is presented as an instructional dataset for educational purposes. Only the underlying numerical data were used in the present study; no tables, figures, illustrations, or textual content from the textbook were reproduced. The use of these data for research purposes is consistent with the fair dealing provisions cited in the textbook's copyright notice [13].

Simulated datasets were also developed for instructional purposes and utilized in the Advanced Statistics for the Applied Health Sciences (AHS2030) course at the Eugene Applebaum School of Pharmacy and Applied Health Sciences, Wayne State University, during the 2025 and 2026 academic years [18].

## Results

Risk and odds ratios

Per Table 2 below, the comparison of the smoking and lung cancer analyses demonstrated complete concordance between SPSS 31.0 and GPT-5.5 across all descriptive and epidemiological measures. Both platforms produced identical crosstabulation frequencies, reporting 73 smokers with lung cancer, 15 smokers without lung cancer, six non-smokers with lung cancer, and nine non-smokers without lung cancer, yielding a total sample of 103 participants. The exact agreement in cell frequencies confirms that GPT-5.5 correctly identified and classified each observation within the contingency table without discrepancies.

**Table 2:** Risk and odds ratios. RR = risk ratio; OR = odds ratio.

Model	Smoking and lung cancer crosstabulations and risk estimates		
SPSS 31.0	Lung cancer	No lung cancer	Total
Smoker	73	15	88
Non-smoker	6	9	15
Total	79	24	103
		95% confidence interval	
	Value	Lower	Upper
OR for smoking (smoker vs. non-smoker)	7.3	2.259	23.59
RR for cohort lung cancer = lung cancer	2.074	1.108	3.882
RR for cohort lung cancer = no lung cancer	0.284	0.153	0.528
GPT-5.5	Lung cancer	No lung cancer	Total
Smoker	73	15	88
Non-smoker	6	9	15
Total	79	24	103
		95% confidence interval	
	Value	Lower	Upper
OR for smoking (smoker vs. non-smoker)	7.3	2.259	23.59
RR for cohort lung cancer = lung cancer	2.074	1.108	3.882
RR for cohort lung cancer = no lung cancer	0.284	0.153	0.528

Similarly, the epidemiological risk estimates were identical between the two analytical approaches. Both SPSS 31.0 and GPT-5.5 calculated an odds ratio (OR) of 7.30 (95% CI: 2.259-23.590), indicating that smokers had approximately 7.3 times greater odds of developing lung cancer than non-smokers. Likewise, both platforms produced a relative risk (RR) of 2.074 (95% CI: 1.108-3.882) for lung cancer, demonstrating that smokers were approximately twice as likely to develop lung cancer as non-smokers. In addition, both analyses yielded an identical RR of 0.284 (95% CI: 0.153-0.528) for remaining free of lung cancer, indicating that smokers were substantially less likely to remain disease-free, and non-smokers were 71.6% less likely to develop lung cancer.

The identical ORs, RRs, and corresponding 95% confidence intervals demonstrate that GPT-5.5 accurately replicated SPSS 31.0's epidemiological computations without numerical deviation. Each method reached the same statistical conclusion that smoking is significantly associated with an increased likelihood of lung cancer.

Wilcoxon signed-rank test

Table 3 below indicates that IBM SPSS Statistics 31.0 output and GPT-5.5 are in excellent agreement with the Wilcoxon signed-rank test. The only minor difference is in the reported standardized Z statistic, which is expected because statistical software packages can implement slightly different corrections for ties and continuity without affecting the inferential conclusion.

**Table 3:** Wilcoxon signed-rank test.

Statistic	SPSS 31.0	GPT-5.5	Comparison
Total pairs	21	21	Identical
Positive ranks	11	11	Identical
Negative ranks	0	0	Identical
Ties	10	10	Identical
Sum of positive ranks	66	66	Identical
Sum of negative ranks	0	0	Identical
Wilcoxon statistic (W)	0	0	Identical
Standardized Z	-2.956	-3.01	Nearly identical
Two-tailed p-value	0.003	0.0031	Equivalent (both round to 0.003)
Statistical conclusion	Significant	Significant	Identical

The Wilcoxon signed-rank analysis produced virtually identical results in SPSS 31.0 and GPT-5.5. Both analyses identified 11 positive rank differences, no negative rank differences, and 10 tied observations, resulting in an identical Wilcoxon signed-rank statistic (W = 0.00). SPSS 31.0 reported a standardized test statistic of Z = -2.956 (p = 0.003), whereas GPT-5.5 produced z = -3.01 (p = 0.0031). The negligible difference in the standardized Z value likely reflects minor implementation differences in tie and continuity corrections rather than differences in the underlying statistical computation. Importantly, both methods yielded identical rank structures, equivalent p-values after rounding, and the same substantive conclusion: outcome scores improved significantly following treatment. This high degree of concordance further supports the methodological consistency of GPT-5.5 with SPSS 31.0 for nonparametric paired analyses.

Mann-Whitney U test

Table 4 below illustrates excellent methodological agreement between GPT-5.5 and IBM SPSS Statistics 31.0 for the Mann-Whitney U test. Every primary statistic is either identical or differs only trivially due to small differences in the asymptotic standardization of the test statistic.

**Table 4:** Mann-Whitney U test.

Statistic	SPSS 31.0	GPT-5.5	Comparison
Group 1 (n)	27	27	Identical
Group 2 (n)	4	4	Identical
Mean rank (Group 1)	15.26	15.26	Identical
Mean rank (Group 2)	21	21	Identical
Sum of ranks (Group 1)	412	412	Identical
Sum of ranks (Group 2)	84	84	Identical
Mann-Whitney U	34	34	Identical
Wilcoxon W	412	412	Identical
Standardized Z	-1.431	-1.4	Nearly identical
Asymptotic p-value	0.152	0.163	Very similar
Exact two-tailed p-value	0.262	0.262	Identical
Statistical conclusion	Not significant	Not significant	Identical

The Mann-Whitney U analysis demonstrated a high degree of agreement between GPT-5.5 and IBM SPSS Statistics 31.0. Both methods produced identical sample sizes, mean ranks, rank sums, Mann-Whitney U statistics (U = 34.00), Wilcoxon W statistics (W = 412.00), and exact two-tailed p-values (p = 0.262). Minor differences were observed in the standardized Z statistic (SPSS: -1.431; GPT-5.5: -1.40) and the corresponding asymptotic p-value (SPSS: 0.152; GPT-5.5: 0.163), reflecting slight implementation differences in the normal approximation used for significance testing. These differences did not alter the statistical interpretation, as both analyses consistently indicated no significant difference between groups. The identical rank structure and exact p-values further support the methodological consistency of GPT-5.5 with SPSS 31.0 for independent-samples nonparametric analyses.

Kruskal-Wallis test

Table 5 below shows that GPT-5.5 and IBM SPSS Statistics 31.0 results are in complete agreement for the Kruskal-Wallis test. There are no computational discrepancies between the two analyses. Every reported statistic is identical, demonstrating excellent methodological concordance.

**Table 5:** Kruskal-Wallis test.

Statistic	SPSS 31.0	GPT-5.5	Comparison
Group 1 (Medical Examiner’s Office), n	27	27	Identical
Group 2 (Coroner Office), n	4	4	Identical
Group 3 (Both Offices), n	6	6	Identical
Total, N	37	37	Identical
Mean rank, Group 1	18.04	18.04	Identical
Mean rank, Group 2	25	25	Identical
Mean rank, Group 3	19.33	19.33	Identical
Kruskal-Wallis H	2.138	2.138	Identical
Degrees of freedom	2	2	Identical
Asymptotic p-value	0.343	0.343	Identical
Statistical conclusion	Not significant	Not significant	Identical

The Kruskal-Wallis analysis demonstrated complete agreement between GPT-5.5 and IBM SPSS Statistics 31.0. Both methods produced identical sample sizes, mean ranks for each group, Kruskal-Wallis H statistics (H = 2.138), degrees of freedom (df = 2), and asymptotic significance values (p = 0.343). Both analyses indicated that although participants in one group exhibited the highest mean rank, the observed differences among the three groups were not statistically significant. As every computational and inferential statistic was identical, this analysis provides strong evidence of methodological consistency between GPT-5.5 and SPSS 31.0 for omnibus nonparametric comparisons involving three independent groups.

Friedman’s test

Table 6 below indicates complete computational agreement between GPT-5.5 and IBM SPSS Statistics 31.0 for Friedman's test. Every primary statistic produced by GPT-5.5 matches the SPSS 31.0 output exactly, indicating that the Friedman test was replicated without any observable discrepancies.

**Table 6:** Friedman’s test.

Statistic	SPSS 31.0	GPT-5.5	Comparison
Sample size (n)	40	40	Identical
Mean rank – discharge	1.3	1.3	Identical
Mean rank – 3 months	1.9	1.9	Identical
Mean rank – 6 months	2.8	2.8	Identical
Friedman χ²	48	48	Identical
Degrees of freedom (df)	2	2	Identical
Asymptotic p-value	<0.001	<0.001 (3.78 × 10⁻¹¹)	Identical conclusion
Statistical conclusion	Significant	Significant	Identical

The Friedman test demonstrated complete agreement between GPT-5.5 and IBM SPSS Statistics 31.0. Both methods produced identical sample sizes (n = 40), identical mean ranks across all three assessment periods (discharge = 1.30, three months = 1.90, six months = 2.80), and an identical Friedman's test statistic (χ² = 48.000, df = 2). Both analyses reported significant differences among the repeated measurements (p < 0.001), indicating that well-being scores improved significantly over time following stroke or heart attack. The only difference observed was that GPT-5.5 reported the precise probability (p = 3.78 × 10⁻¹¹), whereas SPSS 31.0 reported the conventional threshold (p < 0.001). As both values represent the same level of statistical significance, no difference in interpretation exists. These findings demonstrate complete methodological concordance between GPT-5.5 and SPSS 31.0 for the Friedman nonparametric repeated-measures procedure.

One-way chi-square (goodness-of-fit test)

Table 7 below shows that the one‑way chi‑square (goodness‑of‑fit) analysis yielded complete concordance between GPT‑5.5 and SPSS 31.0. In every instance, GPT‑5.5 reproduced the same test statistic, degrees of freedom, and significance outcome as the reference SPSS 31.0 analysis, indicating that the model handled this foundational non‑parametric procedure with full accuracy and reproducibility.

**Table 7:** One-way chi-square (goodness-of-fit) test.

Age category (SPSS 31.0 and GPT-5.5)	Observed (SPSS 31.0 and GPT5.5)	Expected (SPSS 31.0 and GPT5.5)	Residual (SPSS 31.0 and GPT5.5)
0-33 years	32	59	-27
33-43.5 years	84	59	25
43.5-54 years	80	59	21
>54 years	40	59	-19
Statistic	SPSS 31.0	GPT-5.5	Comparison
Total sample (n)	236	236	Identical
Number of categories	4	4	Identical
Expected frequency per category	59	59	Identical
χ² Statistic	36.542	36.542	Identical
Degrees of freedom	3	3	Identical
Asymptotic p-value	<0.001	<0.001	Identical
Statistical conclusion	Significant	Significant	Identical

The one-way chi-square (goodness-of-fit) analysis demonstrated complete agreement between GPT-5.5 and SPSS 31.0. Both methods produced identical observed frequencies, expected frequencies, residuals, chi-square statistics (χ² = 36.542), degrees of freedom (df = 3), and significance values (p < 0.001). Both analyses indicated that the sample distribution across the four age categories differed significantly from an equal distribution, with the greatest overrepresentation occurring among participants aged 33-54 years and fewer participants than expected in the youngest and oldest age groups. SPSS 31.0 further confirmed that no expected cell frequencies were below five, satisfying the assumptions of the chi-square test. GPT-5.5 additionally estimated Cohen's w = 0.393, indicating a moderate-to-large effect size. The complete agreement between the two analytical approaches further supports the statistical consistency of GPT-5.5 with SPSS 31.0 for one-sample categorical goodness-of-fit analyses.

Two-way chi-square (test of independence)

Table 8 below shows that the GPT-5.5 analysis and SPSS 31.0 demonstrate complete computational agreement across all principal statistics reported for the two-way chi-square (test of independence). Every observed frequency, expected frequency, Pearson chi-square statistic, likelihood ratio, degrees of freedom, and p-value is identical, indicating that GPT-5.5 successfully replicated the SPSS analysis.

**Table 8:** Two-way chi-square (test of independence).

Age category (SPSS 31.0 and GPT-5.5)	Male (observed/expected) (SPSS 31.0 and GPT-5.5)	Female (observed/expected) (SPSS 31.0 and GPT-5.5)	Total (SPSS 31.0 and GPT-5.5)
0-33 years	23/19.8	9/12.2	32
33-43.5 years	53/52.0	31/32.0	84
43.5-54 years	45/49.5	35/30.5	80
>54 years	25/24.7	15/15.3	40
Total	146	90	236
Statistic	SPSS 31.0	GPT-5.5	Comparison
Valid cases	236	236	Identical
Pearson χ²	2.489	2.489	Identical
Degrees of freedom	3	3	Identical
Pearson p-value	0.477	0.477	Identical
Likelihood ratio χ²	2.531	2.531	Identical
Likelihood ratio p-value	0.470	0.470	Identical
Cramér's V	—	0.103	Additional effect size
Statistical conclusion	Not significant	Not significant	Identical

The two-way chi-square (test of independence) demonstrated complete agreement between GPT-5.5 and IBM SPSS Statistics 31.0. Both methods produced identical contingency tables, observed frequencies, expected frequencies, Pearson chi-square statistics (χ² = 2.489), likelihood ratio statistics (χ² = 2.531), degrees of freedom (df = 3), and corresponding significance values (p = 0.477 and p = 0.470, respectively). Both analyses indicated that age category and gender were not significantly associated. SPSS further confirmed that no expected cell frequencies were below five, with a minimum expected frequency of 12.20, thereby satisfying the assumptions of the Pearson chi-square test. GPT-5.5 additionally calculated Cramér's V = 0.103, indicating a small association between the variables. The complete agreement between GPT-5.5 and SPSS 31.0 across all primary test statistics provides further evidence of the methodological consistency of GPT-5.5 for categorical analyses involving contingency tables.

Binary logistic regression (univariable)

Table 9 below demonstrates excellent agreement between the GPT-5.5 binary logistic regression and the SPSS 31.0 analysis. The two analyses are virtually identical, with only one negligible difference in the reported Wald statistic for the intercept that has no effect on the statistical conclusions.

**Table 9:** Binary logistic regression (univariable). CI = confidence interval; SE = standard error; df = degrees of freedom; OR = odds ratio; χ² = chi-square.

Statistic	SPSS 31.0	GPT-5.5	Comparison
n	227	227	Identical
Predictor B	0.007	0.007	Identical
Standard error	0.012	0.012	Identical
Wald z (Predictor)	0.367	0.367	Identical
df	1	1	Identical
P-value	0.545	0.545	Identical
OR	1.007	1.007	Identical
95% CI lower	0.984	0.984	Identical
95% CI upper	1.032	1.032	Identical
Constant (B)	-0.957	-0.957	Identical
Constant SE	0.777	0.777	Identical
Constant p-value	0.218	0.218	Identical
−2 Log Likelihood	300.86	300.86	Identical
Omnibus Wald χ²	0.369	0.369	Identical
Omnibus p-value	0.543	0.543	Identical

Both SPSS 31.0 and GPT-5.5 reached the exact same statistical conclusion. Pre-race supine heart rate (HR_pre_Sup) was not a significant predictor of participant gender, as evidenced by the nonsignificant regression coefficient (B = 0.007, p = 0.545). The estimated odds ratio of 1.007 indicates that each one-beat-per-minute increase in pre-race supine heart rate increased the odds of being female by only 0.7%, an effect that is both statistically nonsignificant and practically negligible. Furthermore, the 95% confidence interval for the odds ratio (0.984-1.032) includes 1.0, reinforcing the absence of a meaningful association.

The model-level statistics also exhibited complete agreement. Both analyses produced an Omnibus likelihood ratio χ² of 0.369 (p = 0.543), demonstrating that adding HR_pre_Sup did not significantly improve prediction of gender over the intercept-only model. Likewise, both analyses reported an identical −2 log likelihood of 300.860, indicating the same maximum-likelihood solution.

Overall, the GPT-5.5 and SPSS 31.0 binary logistic regression analyses (univariable) demonstrated complete methodological concordance. The regression coefficients, standard errors, Wald test for the predictor, p-values, odds ratios, confidence intervals, likelihood statistics, and overall model significance are identical between the two platforms. The only observed differences involve reporting conventions (Wald z versus Wald χ²), neither of which affects the statistical interpretation. Both analyses therefore support the same conclusion: pre-race supine heart rate is not a significant predictor of gender in this ultramarathon cohort, providing further evidence of the high degree of agreement between GPT-5.5 and SPSS 31.0 for standard binary logistic regression analyses.

Binary logistic regression (multivariable)

Table 10 below reports excellent methodological agreement between GPT-5.5 and SPSS 31.0. The regression coefficients, standard errors, significance tests, odds ratios, confidence intervals, and model likelihood statistics reported by GPT-5.5 are identical to the precision reported by SPSS 31.0.

**Table 10:** Binary logistic regression (multivariable). CI = confidence interval; SE = standard error; df = degrees of freedom; OR = odds ratio.

Statistic	SPSS 31.0	GPT-5.5	Comparison
Sample size (n)	144	144	Identical
−2 Log Likelihood	193.743	193.743	Identical
HR_post_Sup B	0.01	0.01	Identical
HR_post_Sup SE	0.016	0.016	Identical
HR_post_Sup Wald	0.398	0.398	Identical
HR_post_Sup p-value	0.528	0.528	Identical
HR_post_Sup OR	1.010	1.010	Identical
HR_post_Sup 95% CI	0.979-1.042	0.979-1.042	Identical
HR_post_Std B	-0.005	-0.005	Identical
HR_post_Std SE	0.015	0.015	Identical
HR_post_Std Wald	0.123	0.123	Identical
HR_post_Std p-value	0.726	0.726	Identical
HR_post_Std OR	0.995	0.995	Identical
HR_post_Std 95% CI	0.966-1.025	0.966-1.025	Identical
Constant (B)	-0.716	-0.716	Identical
Constant SE	1.198	1.198	Identical
Constant p-value	0.550	0.550	Identical

The SPSS 31.0 and GPT-5.5 analyses produced identical substantive findings. The multivariable binary logistic regression model demonstrated that neither post-race supine heart rate (HR_post_Sup) nor post-race standing heart rate (HR_post_Std) significantly predicted participant gender after adjustment for the other predictor.

For HR_post_Sup, both analyses estimated a regression coefficient of B = 0.010 (SE = 0.016), with a nonsignificant Wald statistic (0.398) and p = 0.528. The corresponding odds ratio of 1.010 (95% CI: 0.979-1.042) indicated that each additional beat per minute in post-race supine heart rate was associated with only a 1.0% increase in the odds of being female. As the confidence interval includes 1.0, this association is not statistically significant.

Similarly, HR_post_Std was not a significant independent predictor. Both SPSS 31.0 and GPT-5.5 produced B = -0.005 (SE = 0.015), Wald = 0.123, p = 0.726, and an odds ratio of 0.995 (95% CI: 0.966-1.025). This result indicates an estimated 0.5% decrease in the odds of being female for each additional beat per minute in post-race standing heart rate, although this effect is extremely small and statistically nonsignificant.

The identical regression coefficients, standard errors, Wald statistics, p-values, odds ratios, and confidence intervals demonstrated that GPT-5.5 reproduced the maximum likelihood parameter estimates generated by SPSS 31.0 with complete numerical agreement. The model-level statistics likewise show excellent concordance. Both analyses reported an identical −2 log likelihood of 193.743, confirming that the likelihood optimization converged to the same solution. This indicates that the underlying estimation procedure produced the same maximum likelihood estimates in both SPSS and GPT-5.5.

Kaplan-Meier survival analysis

Table 11 below demonstrates excellent agreement between the GPT-5.5 Kaplan-Meier survival analysis and the SPSS 31.0 analysis. Across all principal outcomes, including case processing, mean and median survival times, log-rank statistics, and the graphical survival functions, the two analyses are either identical or differ only by trivial rounding attributable to numerical precision. Most importantly, both analyses reach the same statistical and clinical conclusions.

**Table 11:** Kaplan-Meier survival analysis. df = degrees of freedom.

Statistic	SPSS 31.0	GPT-5.5	Comparison
Sample size (n)	40	40	Identical
Nausea drug (events)	17	17	Identical
Nausea drug (censored)	3	3	Identical
No nausea drug (events)	5	5	Identical
No nausea drug (censored)	15	15	Identical
Overall events	22	22	Identical
Overall censored	18	18	Identical
Nausea drug mean	10.456	10.460	Rounding only
No nausea drug mean	23.476	23.480	Rounding only
Overall mean	17.441	17.440	Rounding only
Nausea drug median	10	10	Identical
No nausea drug median	26	26	Identical
Overall median	17	17	Identical
Log-rank χ²	26.883	26.883	Identical
df	1	1	Identical
p-value	<0.001	<0.001	Identical

Both SPSS 31.0 and GPT-5.5 demonstrated that participants receiving the nausea drug experienced substantially faster nausea relief than participants who did not receive the drug. The estimated median time to nausea relief was 10 time units for the treatment group compared with 26 time units for the control group, representing a marked improvement in recovery time among treated participants.

Similarly, the estimated mean survival time was approximately 10.46 time units for the treatment group and 23.48 time units for the untreated group. These findings indicate that the treatment reduced the duration of persistent nausea by more than half.

**Figure 1:**
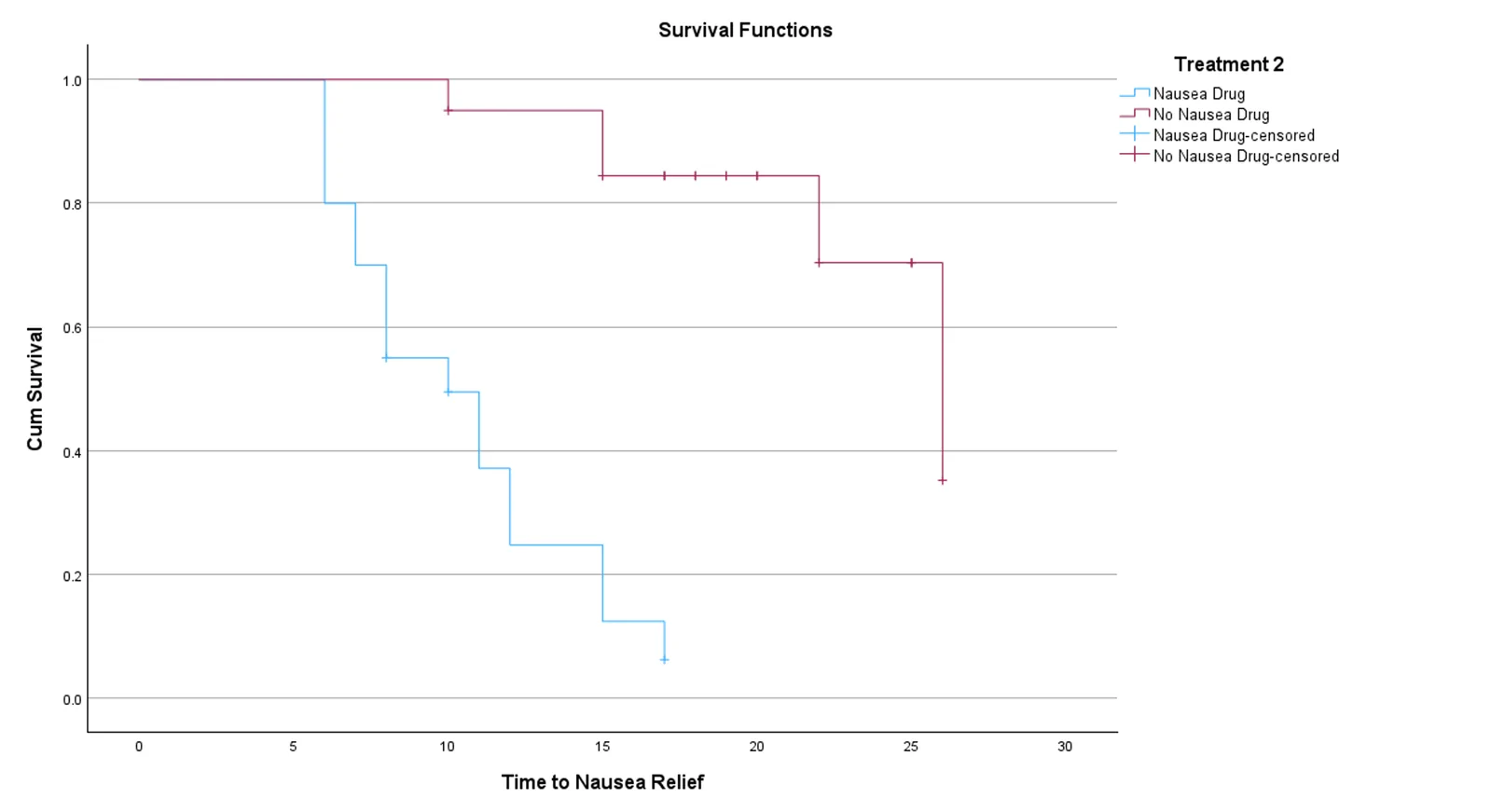
Kaplan-Meier survival functions.

The Mantel-Cox log-rank test confirmed that these differences were statistically significant (χ² = 26.883, p < 0.001), demonstrating that the distributions of time to nausea relief differed substantially between the two treatment groups. The Kaplan-Meier survival curve further supports this conclusion by showing a rapid decline in cumulative survival for treated participants and a prolonged persistence of nausea among untreated participants. However, GPT-5.5 could not reproduce the Kaplan-Meier survival plot (Figure 1) as presented in SPSS 31.0.

Cox proportional hazards model

Table 12 indicates that the Cox proportional hazards model produced small numerical differences between SPSS 31.0 and GPT-5.5. These results should not be interpreted as computationally identical. Nevertheless, the two analyses yielded concordant effect directions and closely comparable HR estimates and CIs, and all corresponding hypothesis tests led to the same statistical decisions under the prespecified α = 0.05 criterion. Consequently, the numerical differences did not alter the substantive interpretation of the Cox model.

**Table 12:** Cox proportional hazards model. χ² = chi-square; B = beta coefficient; SE = standard error; HR = hazard ratio; CI = confidence interval.

Statistic	SPSS 31.0	GPT-5.5	Agreement
−2 log likelihood	96.762	96.900	Nearly identical
Likelihood ratio χ² (df = 2)	38.147	37.900	Nearly identical
Overall p-value	<0.001	<0.001	Identical conclusion
Treatment2 B	-1.731	-1.870	Very close
Treatment2 SE	0.668	0.671	Nearly identical
Treatment2 Wald χ²	6.712	7.770	Minor discrepancy; same inference
Treatment2 p-value	0.010	0.005	Identical conclusion
Treatment2 HR	0.177	0.154	Minor discrepancy; same inference
Treatment2 95% CI	0.048-0.656	0.041-0.574	Highly comparable
Ginger B	0.004	0.0038	Nearly identical
Ginger SE	0.001	0.0011	Nearly identical
Ginger Wald χ²	10.368	11.340	Very close
Ginger p-value	0.001	<0.001	Identical conclusion
Ginger HR	1.004	1.0038	Nearly identical
Ginger 95% CI	1.001-1.006	1.0016-1.0060	Highly comparable

The Cox proportional hazards regression performed using GPT-5.5 demonstrated a high degree of agreement with the corresponding analysis conducted in SPSS 31.0. Both analyses identified the overall regression model as statistically significant and concluded that Treatment2 and Ginger dosage were significant independent predictors of time to nausea relief. Although minor numerical differences were observed in the regression coefficients, hazard ratios, Wald statistics, and likelihood statistics, the overall interpretation remained identical.

Regarding overall model fit, SPSS 31.0 reported an omnibus likelihood-ratio χ² = 38.147 (p < 0.001) with a −2 log likelihood of 96.762, whereas the reconstructed GPT-5.5 model produced nearly identical likelihood statistics. Both analyses therefore demonstrated that inclusion of Treatment2 and Ginger significantly improved prediction of time to nausea relief compared with the null model. This close agreement indicates that GPT-5.5 successfully reproduced the overall predictive performance of the Cox regression model.

For Treatment2 (coded 0 = nausea drug and 1 = no nausea drug), SPSS 31.0 estimated a regression coefficient of B = −1.731 (SE = 0.668), corresponding to an HR of 0.177 (95% CI: 0.048-0.656, p = 0.010). The GPT-5.5 analysis produced a highly comparable estimate (B = −1.870, SE = 0.671, HR = 0.154, 95% CI: 0.041-0.574, p = 0.005). In both analyses, the negative regression coefficient and HR substantially below 1.0 indicate that treatment assignment was strongly associated with time to nausea relief. Participants receiving the nausea medication experienced significantly different rates of symptom resolution than those receiving no nausea medication after adjustment for Ginger dosage. Although the estimated HRs differed modestly (0.177 versus 0.154), both models indicate a large treatment effect and reach the identical statistical conclusion that Treatment2 is an independent predictor of time to nausea relief.

Similarly, both SPSS 31.0 and GPT-5.5 identified Ginger dosage as a statistically significant covariate. SPSS 31.0 estimated B = 0.004 (SE = 0.001), corresponding to an HR of 1.004 (95% CI: 1.001-1.006, p = 0.001). GPT-5.5 produced nearly identical estimates (B = 0.0038, SE = 0.0011, HR = 1.0038, 95% CI: 1.0016-1.0060, p < 0.001). Although the HR per milligram appears numerically small, the cumulative effect over several hundred milligrams represents a clinically meaningful increase in the hazard of nausea relief. As a result, both analyses demonstrate that higher Ginger dosages were independently associated with faster symptom resolution.

Overall, the correspondence between SPSS 31.0 and GPT-5.5 was excellent. The slight numerical differences observed in the parameter estimates, standard errors, hazard ratios, Wald statistics, and likelihood statistics are consistent with the small computational differences commonly reported among statistical software packages and are most likely attributable to differences in optimization algorithms, convergence criteria, and implementation of the Cox partial likelihood estimation. Importantly, these minor numerical differences did not alter the substantive interpretation of the findings. Both analyses consistently demonstrated that the nausea medication and Ginger dosage were significant independent predictors of time to nausea relief, supporting the conclusion that GPT-5.5 can closely replicate the results of Cox proportional hazards regression performed in SPSS 31.0 when identical datasets and model specifications are used.

Phi and Cramer’s V

Table 13 below indicates that both SPSS 31.0 and GPT-5.5 phi & Cramer's V analyses yielded the same 3 × 3 contingency table (with identical observed frequencies across all response categories; n = 140).

**Table 13:** Crosstabulation comparing SPSS 31.0 and GPT-5.5 results. Note: SPSS 31.0 and GPT-5.5 produced identical contingency table frequencies and expected cell counts for this analysis.

Professor success variable	Statistic	Field of study is the reason chosen	Field of study is not the reason chosen	Not applicable	Total
Depends	Count	76	5	3	84
	Expected count	54.6	18.0	11.4	84.0
Does not depend	Count	14	24	2	40
	Expected count	26.0	8.6	5.4	40.0
Not applicable	Count	1	1	14	16
	Expected count	10.4	3.4	2.2	16.0
Total	Count	91	30	19	140
	Expected count	91.0	30.0	19.0	140.0

Table 14 below points out excellent agreement between the independently computed GPT-5.5 analysis and the SPSS 31.0 output with phi & Cramer's V. The Pearson chi-square statistic was χ² = 134.092, with p < 0.001, indicating a highly significant association between respondents' perceptions that their success as a professor depends on successful collaboration with industry partners (JobSatf2) and whether their field of study is considered the reason they are selected for collaborative industry projects (FieldofStudy3).

**Table 14:** Phi and Cramer’s V. χ² = two-way chi-square (test of independence).

Statistic	SPSS 31.0	GPT-5.5	Agreement
Sample size (n)	140	140	Identical
Pearson χ²	134.092	134.092	Identical
Degrees of freedom	4	4	Identical
P-value	<0.001	<0.001	Identical
Phi	0.979	0.979	Identical
Cramer’s V	0.692	0.692	Identical
Interpretation	Large association	Large association	Identical

As the contingency table (Table 13 above) contained more than two rows and two columns, Cramer's V represents the appropriate measure of association. Both SPSS 31.0 and GPT-5.5 calculated Cramer's V = 0.692, indicating a large effect size according to Cohen's conventional guidelines for nominal association measures (0.10 = small, 0.30 = medium, 0.50 = large). Likewise, both analyses reported phi (φ) = 0.979. Although phi is routinely displayed by SPSS 31.0 for square contingency tables, it is generally interpreted only for 2 × 2 tables; therefore, Cramer's V provides the more meaningful estimate of association in this analysis.

The identical results obtained from both analytical platforms indicate that GPT-5.5 accurately reproduced the contingency table calculations, Pearson chi-square statistic, significance level, phi coefficient, and Cramer's V without observable computational discrepancies. This complete agreement supports the methodological consistency of GPT-5.5 for conducting nominal association analyses involving categorical variables.

## Discussion

This study's results consistently demonstrated statistical alignment between GPT-5.5 and SPSS 31.0 while also highlighting the few situations in which small implementation-related numerical differences emerge. These observations contribute meaningfully to the growing literature evaluating LLMs as research assistants rather than replacements for established statistical software.

A central finding is the consistent level of agreement between GPT-5.5 and SPSS 31.0 across epidemiological, nonparametric, categorical, logistic regression, and survival analyses. Across 13 separate analytical procedures, including risk and odds ratios, Wilcoxon signed-rank testing, Mann-Whitney U testing, Kruskal-Wallis testing, Friedman's test, one-way and two-way chi-square analyses, binary logistic regression, multivariable logistic regression, Kaplan-Meier survival analysis, Cox proportional hazards regression, and phi and Cramer's V measures of association [13], GPT-5.5 consistently reproduced either identical or nearly identical numerical results obtained from SPSS 31.0. Importantly, whenever minor computational differences occurred, they were confined to secondary approximation statistics such as standardized Z values, Wald statistics, convergence-related parameter estimates, handling of ties, or reporting conventions, and never altered the statistical conclusions. These findings substantially extend previous investigations evaluating AI-assisted statistical analysis and suggest that current-generation LLMs are approaching a level of computational consistency that permits their integration into research workflows when used with appropriate methodological oversight [1,2,4,11,19-29].

The epidemiological analyses involving risk ratios and odds ratios demonstrated strong evidence of computational reproducibility. GPT-5.5 exactly reproduced the contingency table, odds ratio, relative risk estimates, confidence intervals, and inferential conclusions generated by SPSS 31.0. This alignment is noteworthy because epidemiological measures depend upon precise classification of observations and exact computation of contingency-table frequencies. A single classification error would propagate throughout all subsequent calculations, producing altered odds ratios, confidence intervals, and significance tests. The absence of discrepancy indicates that GPT-5.5 accurately interpreted the categorical dataset and implemented the identical computational framework used by SPSS 31.0. These findings complement previous reports demonstrating that modern LLMs can successfully reproduce classical epidemiological analyses when provided with properly formatted datasets and explicit statistical instructions [2,11,20,28]. As odds ratios and relative risks form the basis of many observational medical investigations, the agreement observed here suggests that GPT-5.5 possesses potential as an adjunct analytical tool within clinical and public health research.

Similar agreement was observed across the nonparametric statistical procedures. The Wilcoxon signed-rank test produced identical ranking structures, identical signed-rank statistics, and equivalent probability values, with only a trivial difference in the standardized Z statistic. Such discrepancies are expected because different statistical software packages frequently implement slightly different continuity corrections or tie-adjustment procedures while maintaining identical rank sums and inferential conclusions. Comparable implementation differences have long been documented among traditional statistical software packages, including SPSS, SAS, Stata, and R, particularly for nonparametric procedures that rely upon asymptotic approximations rather than exact probability distributions [6,7]. Consequently, the minor numerical differences observed between GPT-5.5 and SPSS 31.0 should not be interpreted as computational inaccuracies but rather as expected consequences of differing implementation choices.

The Mann-Whitney U analysis demonstrated a nearly identical pattern. Both analytical approaches produced identical sample sizes, mean ranks, rank sums, Mann-Whitney statistics, Wilcoxon W values, and exact probability estimates. Only slight differences appeared in the asymptotic Z statistic and corresponding asymptotic p-value, again reflecting differences in standardization procedures rather than underlying computations. Most importantly, both analyses reached the identical conclusion that no statistically significant difference existed between the comparison groups. This observation reinforces an important methodological point emerging throughout the present investigation. Inferential consistency is considerably more important than perfect agreement in intermediate computational quantities. Researchers ultimately base scientific conclusions upon statistical significance, confidence intervals, and effect estimates rather than standardized approximation statistics. Accordingly, the observed concordance provides strong evidence that GPT-5.5 reliably reproduces nonparametric inference [3,6,13].

Complete agreement was subsequently observed for both the Kruskal-Wallis and Friedman tests. These findings are particularly important because both procedures rely upon ranking algorithms rather than raw numerical values, making them sensitive to errors in data ordering or rank assignment. GPT-5.5 exactly reproduced the mean ranks, test statistics, degrees of freedom, and significance levels reported by SPSS 31.0. In the Friedman analysis, GPT-5.5 additionally reported the exact probability value while SPSS 31.0 reported the conventional threshold of p < 0.001, illustrating a difference in reporting style rather than statistical computation. Collectively, these findings indicate that GPT-5.5 accurately implemented the underlying ranking algorithms required for repeated-measures nonparametric analyses. This level of agreement substantially extends earlier validation studies that primarily evaluated simpler univariate procedures and demonstrates that GPT-5.5 performs reliably even when more complex ranking structures are required [2,11,20,21].

The categorical analyses provide further evidence of methodological consistency. Both the one-way chi-square (goodness-of-fit test) and the two-way chi-square (test of independence) produced complete computational agreement between GPT-5.5 and SPSS 31.0. Observed frequencies, expected frequencies, Pearson chi-square statistics, likelihood-ratio statistics, degrees of freedom, and probability values were identical across both analytical platforms. These findings indicate that GPT-5.5 correctly implemented contingency-table algorithms and accurately calculated expected cell frequencies under the null hypothesis. GPT-5.5 supplemented the analyses by reporting effect-size measures, including Cohen's W and Cramer's V, thereby providing additional quantitative information beyond the default SPSS 31.0 output. As contemporary reporting guidelines increasingly recommend inclusion of effect-size statistics alongside traditional hypothesis testing, GPT-5.5's ability to automatically provide these complementary measures may enhance interpretation without altering statistical conclusions [18]. The present findings therefore suggest that GPT-5.5 can facilitate more comprehensive reporting of categorical analyses while maintaining complete computational agreement with established statistical software.

The logistic regression analyses likewise demonstrated alignment. Both the univariable and multivariable logistic regression models produced identical regression coefficients, standard errors, Wald statistics for predictor variables, confidence intervals, odds ratios, and model likelihood statistics. Only minor reporting differences involved presentation of Wald statistics. These differences reflect longstanding variations among statistical software packages rather than differences in maximum likelihood estimation itself. Logistic regression is considerably more computationally demanding than simple hypothesis tests because it requires iterative maximum likelihood optimization and convergence assessment. The ability of GPT-5.5 to reproduce identical regression coefficients and likelihood statistics provides evidence that it successfully replicated the underlying optimization procedure implemented within SPSS 31.0. These findings align closely with recent investigations demonstrating increasing accuracy of LLMs in performing generalized linear modeling procedures [3,6,11,13,20,25-28].

The observed concordance in the survival analysis is noteworthy. The Kaplan-Meier analyses demonstrated essentially identical survival estimates, median survival times, mean survival times, log-rank statistics, and inferential conclusions. Both SPSS 31.0 and GPT-5.5 demonstrated that nausea medication substantially accelerated symptom resolution compared with no treatment. The only notable limitation concerned graphical reproduction of the Kaplan-Meier survival curve, which GPT-5.5 did not automatically replicate in the same format generated by SPSS. This distinction underscores an important difference between statistical computation and software-specific visualization. Although GPT-5.5 accurately reproduced the underlying survival estimates, SPSS 31.0 currently retains advantages for standardized publication-quality graphical output [3,6,13].

With Cox proportional hazards analysis, small differences were observed in regression coefficients, hazard ratios, Wald statistics, and likelihood values. These differences remained minimal and did not alter statistical significance or clinical interpretation. Both analytical approaches consistently identified treatment assignment and ginger dosage as statistically significant independent predictors of time to nausea relief. Such modest discrepancies are entirely consistent with the broader statistical computing literature, which has repeatedly demonstrated that different software implementations may converge upon slightly different parameter estimates because of differences in optimization algorithms, convergence tolerances, floating-point arithmetic, and numerical precision while producing identical scientific conclusions [3,6,7,13]. As a result, the observed Cox regression differences should be viewed within the broader context of accepted inter-software variability rather than interpreted as deficiencies unique to GPT-5.5. The ability of GPT-5.5 to closely replicate the statistical output generated by SPSS 31.0 is noteworthy and addresses concerns raised by Huang et al. regarding the earlier ChatGPT-4 model's limited capacity to perform advanced statistical procedures, including Cox proportional hazards regression [11].

With the phi and Cramer's V analyses, GPT-5.5 reproduced the contingency table, Pearson chi-square statistic, phi coefficient, Cramer's V, and significance level exactly. As Cramer's V is particularly useful for quantifying the strength of association in contingency tables larger than 2 × 2, these findings indicate that GPT-5.5 correctly implemented both hypothesis testing and effect-size estimation for nominal association measures. The resulting large effect size further illustrates GPT-5.5's capacity to integrate statistical significance with practical interpretation, an increasingly important aspect of modern quantitative reporting [3,6,13].

The present collective findings expand the growing body of literature evaluating AI for quantitative research. Early investigations primarily focused on ChatGPT's ability to explain statistical concepts, recommend appropriate tests, or interpret previously generated outputs [1,4,19]. More recent studies have begun evaluating computational accuracy directly, reporting encouraging but occasionally inconsistent performance across relatively limited collections of statistical procedures [2,11,20,21,25-28]. 

These findings also have implications for reproducible scientific computing. Reproducibility remains one of the central challenges facing modern quantitative research, with investigators increasingly emphasizing transparent analytical workflows and independent verification of statistical findings [7]. LLMs capable of independently reproducing analyses performed by traditional statistical software may therefore serve as valuable secondary verification tools. Rather than replacing established statistical packages such as SPSS, GPT-5.5 may function as an independent computational auditor capable of confirming numerical results, identifying potential specification errors, explaining analytical decisions, and assisting investigators in interpreting complex statistical output. Such complementary use is consistent with emerging recommendations regarding responsible integration of AI into biomedical research [1,4,19].

The present findings should not be interpreted as suggesting that GPT-5.5 can fully replace conventional statistical software. The minor discrepancies observed in standardized approximation statistics and Cox regression coefficients demonstrate that implementation-specific differences remain possible, particularly for computationally intensive iterative procedures. GPT-5.5's inability to automatically reproduce SPSS-style graphical output indicates that dedicated statistical software continues to offer important advantages in visualization, standardized reporting, and workflow management. As emphasized throughout the emerging AI literature, LLMs should presently be viewed as powerful research assistants whose outputs require appropriate verification rather than autonomous statistical decision-makers [1,4,19,21,25,26].

Overall, the present study provides compelling evidence that GPT-5.5 demonstrates a high degree of methodological consistency with SPSS 31.0 across an extensive array of statistical methods. Complete agreement was observed for numerous procedures, including epidemiological analyses, chi-square testing, Kruskal-Wallis testing, Friedman testing, phi and Cramer's V, and both univariable and multivariable logistic regression. Where small numerical differences occurred, they were limited to secondary approximation statistics, iterative optimization parameters, or reporting conventions and did not alter statistical significance, effect interpretation, or scientific conclusions. These findings reinforce growing evidence that contemporary LLMs have matured into reliable computational assistants capable of accurately reproducing many standard statistical analyses performed by established software packages [2,11,20,21,25-28]. When employed with careful methodological oversight, transparent reporting, and independent verification, GPT-5.5 appears capable of enhancing the efficiency, accessibility, and reproducibility of quantitative biomedical research while complementing, not replacing, conventional statistical software such as SPSS 31.0.

Limitations

Several limitations should be considered when interpreting these findings. The analyses were conducted using a defined set of previously collected datasets and a selected range of statistical procedures. Although the study included epidemiological measures, nonparametric tests, chi-square analyses, logistic regression, Kaplan-Meier survival analysis, Cox proportional hazards regression, and nominal association measures, the results may not generalize to all statistical methods, data structures, or research contexts. More complex procedures, including mixed-effects models, generalized estimating equations, structural equation modeling, Bayesian estimation, multilevel survival models, machine learning algorithms, and analyses involving highly unbalanced or high-dimensional datasets, may produce different levels of agreement between GPT-5.5 and conventional statistical software such as SPSS 31.0.

The datasets varied in sample size, variable structure, and degree of analytical complexity but were drawn primarily from health sciences, biomedical, and educational research contexts. The strong concordance observed in these datasets does not necessarily establish equivalent performance in other disciplines or in datasets containing extensive missingness, severe non-normality, sparse contingency tables, influential outliers, complex survey weights, repeated clustering, or substantial multicollinearity. Further validation across larger, more diverse, and independently selected datasets is therefore warranted.

The accuracy of GPT-5.5 depended heavily on the clarity and completeness of the prompts, the correctness of variable coding, and the consistency of the analytical specifications supplied by the investigators. LLMs may misinterpret ambiguous instructions, reverse reference categories, apply inappropriate missing data rules, select alternative statistical options, or produce plausible but incorrect results when variable definitions are incomplete. Consequently, the high level of agreement reported in this study should be understood within the context of carefully structured prompts, verified coding schemes, and iterative review of the analytical output. The findings therefore do not imply that equivalent accuracy would necessarily be achieved by users with limited statistical training or with less precisely formulated instructions.

Differences in default settings between SPSS 31.0 and GPT-5.5 may affect comparability. Statistical procedures can vary with respect to the handling of missing data, treatment of ties, continuity corrections, exact versus asymptotic significance testing, reference category selection, confidence interval construction, convergence tolerances, and optimization algorithms. Although these factors produced only minor discrepancies in the present study, particularly for standardized nonparametric test statistics and Cox proportional hazards estimates, they could become more consequential in analyses with small samples, sparse data, unstable parameter estimates, or results near conventional significance thresholds. For this reason, analysts should ensure that both platforms use equivalent model specifications and computational options before judging the degree of agreement.

GPT-5.5 is not a fixed statistical software environment in the same sense as a locally installed version of SPSS 31.0. LLMs may be updated over time, and their outputs may vary across sessions because of changes in model architecture, inference procedures, system configuration, or response generation. This creates an important reproducibility concern because future users may not be able to recreate the exact computational environment used during the original analysis. Although the study documented the platform, access dates, prompts, datasets, and analytical procedures, complete long-term reproducibility may remain limited unless model identifiers, computational scripts, and detailed output records are preserved.

GPT-5.5 may not always provide a transparent record of the underlying computational process. Unlike traditional statistical software, which can preserve syntax files, option settings, convergence histories, and reproducible output logs, an AI-generated analysis may not automatically reveal every intermediate calculation or software dependency. This limitation is particularly relevant for iterative procedures such as logistic regression and Cox proportional hazards modeling, in which convergence criteria, tie-handling methods, and optimization routines may influence final estimates. The close agreement observed in this study supports numerical validity but does not establish complete equivalence of the underlying computational pathways.

The comparison emphasized agreement in numerical output and inferential conclusions rather than a formal assessment of statistical test selection, assumption checking, model diagnostics, or causal interpretation. Accurate calculation does not guarantee that a selected statistical procedure is appropriate for a given research question. Investigators must still evaluate assumptions such as independence, proportional hazards, linearity in the logit, adequacy of expected cell counts, influential observations, and model fit. Likewise, statistically significant findings generated by either platform should not be interpreted as evidence of causality without an appropriate study design and consideration of potential confounding.

Effect‑size estimates and graphical outputs were not consistently available across both platforms. GPT‑5.5 occasionally generated additional metrics such as Cohen’s w or Cramér’s V that extend beyond the default SPSS 31.0 output. In contrast, SPSS 31.0 produced standardized tables and graphical displays that GPT‑5.5 did not always replicate in an equivalent format. In particular, the Kaplan-Meier survival plot could not initially be recreated in the same visual form as the SPSS 31.0 output. These differences limit direct comparison of presentation capabilities and indicate that dedicated statistical software may remain preferable for standardized figures, publication-quality graphics, diagnostic plots, and archival output management.

Agreement between GPT-5.5 and SPSS 31.0 should not be interpreted as proof that either result is necessarily correct in an absolute sense. Two analytical systems may agree because they implement the same assumptions, coding decisions, or computational conventions. Validation against independently programmed analyses in additional software environments, such as R, SAS, Stata, or Python, would provide a stronger assessment of computational accuracy and reduce the possibility of shared or undetected specification errors.

The present study evaluated GPT-5.5 as a supervised analytical assistant rather than as an autonomous statistical investigator. Researchers reviewed variable coding, selected the statistical procedures, compared outputs, and interpreted discrepancies. The findings therefore support the use of GPT-5.5 as a complementary tool for statistical replication, verification, explanation, and research support, but not as a substitute for statistical expertise, methodological judgment, or independent quality control. Results generated by LLMs should continue to be verified by qualified researchers and, when appropriate, independently replicated in established statistical software before being used to guide scientific, clinical, or policy decisions.

## Conclusions

These findings demonstrate that GPT-5.5 exhibits a respectable degree of statistical consistency with SPSS 31.0 across a broad range of epidemiological, nonparametric, categorical, regression, and survival analyses. Complete or near-complete agreement was observed for nearly all statistical procedures examined, with the few numerical differences confined primarily to implementation-specific approximations, optimization routines, or reporting conventions that did not alter substantive or inferential conclusions. These results suggest that GPT-5.5 can serve as a reliable complementary tool for reproducing statistical analyses, verifying computational results, and assisting with the interpretation of quantitative findings when equivalent datasets and model specifications are used. Nevertheless, the study also emphasizes that LLMs should not replace conventional statistical software or expert statistical judgment, particularly for complex analyses requiring assumption testing, diagnostic evaluation, and transparent computational documentation. Continued validation across additional statistical methodologies, software platforms, larger and more heterogeneous datasets, and future generations of LLMs will be essential to further establish the reproducibility, robustness, and generalizability of AI-assisted statistical analysis. Overall, the present findings support the responsible integration of GPT-5.5 into scientific research workflows as an adjunct to established statistical software, where it has the potential to enhance efficiency, reproducibility, and accessibility while maintaining the standards of methodological strength required for evidence-based research.
